# Maternal betel quid use during pregnancy and child growth: a cohort study from rural Bangladesh

**DOI:** 10.1080/16549716.2024.2375829

**Published:** 2024-07-09

**Authors:** Mohammad Redwanul Islam, Shaki Aktar, Jesmin Pervin, Syed Moshfiqur Rahman, Monjur Rahman, Anisur Rahman, Eva-Charlotte Ekström

**Affiliations:** a Global Health and Migration Unit, Department of Women’s and Children’s Health, Uppsala University, Uppsala, Sweden; b Maternal and Child Health Division, International Centre for Diarrhoeal Disease Research, Dhaka, Bangladesh

**Keywords:** Betel quid, areca nut, betel quid use during pregnancy, child growth, rural Bangladesh

## Abstract

**Background:**

Chewing betel quid (BQ) – a preparation commonly containing areca nut and slaked lime wrapped in betel leaf – is entrenched in South Asia. Although BQ consumption during pregnancy has been linked to adverse birth outcomes, its effect on postnatal growth remains largely unexplored.

**Objective:**

We examined the associations of BQ use during pregnancy with children’s height-for-age and body mass index-for-age z-scores (HAZ and BAZ, respectively) and fat and fat-free mass along with sex-based differences in association in rural Bangladesh.

**Methods:**

With a prospective cohort design, we assessed BQ use among mothers enrolled in the Preterm and Stillbirth Study, Matlab (*n* = 3140) with a structured questionnaire around early third trimester. Children born to a subset of 614 women (including 134 daily users) were invited to follow-up between October 2021 and January 2022. HAZ and BAZ were calculated from anthropometric assessment, and fat and fat-free mass were estimated using bioelectric impedance. Overall and sex-specific multiple linear regression models were fitted.

**Results:**

Growth data were available for 501 children (mean age 4.9 years): 43.3% of them were born to non-users, 35.3% to those using prior to or less-than-daily during the survey, and 21.3% to daily users. No statistically significant associations were observed after adjusting for sex, parity, maternal height and education, and household wealth.

**Conclusions:**

There was no effect of BQ use during pregnancy on postnatal growth in this study. Longitudinal studies following up those born to heavy users beyond childhood are warranted for capturing long-term implications of prenatal BQ exposure.

## PAPER CONTEXT

- **Main findings**: In this cohort study, no association was observed between maternal betel quid use during pregnancy and children’s growth around five years of age.
- **Added knowledge**: Although catch-up growth among those born to heavy users may have attenuated any negative impact of prenatal exposure to betel quid on postnatal growth, such catch-up growth often involves greater acquisition and a more centralized distribution of body fat and insulin resistance later in life; leading to a potential heightening of cardiometabolic risk.
- **Global health impact for policy and action**: Given that betel quid consumption during pregnancy remains socially acceptable across south and south-east Asia, this study highlights the need for following up those born to betel quid users beyond childhood for capturing long-term health implications of prenatal betel quid exposure

## Background

The practice of chewing betel quid (BQ) is widely prevalent in south and south-east Asia, the Pacific islands, and high-income countries with migrant populations from these regions [1]. BQ, known as ‘paan’ in Bangla and Hindi, is a chewable wrap ready for placing and retaining in the mouth. The preparation of BQ varies across regions and by user preferences. Nonetheless, it invariably contains areca nut and slaked lime (calcium hydroxide) – with or without catechu, cured tobacco leaves and various flavorings – enclosed in a leaf of the vine *Piper betle* (betel leaf). The core component, areca nut, is the fibrous seed inside the fruit of *Areca catechu* (oriental palm) [1,2]. An estimate published in 2002 [3] put the number of areca-nut users at 600 million globally. Moreover, arecoline, the predominant alkaloid in areca nut, has been cited as the fourth most consumed psychoactive substance following nicotine, ethanol and caffeine [4]. Thus, BQ chewing tends to be addictive [5].

Multiple studies have associated BQ use with oral cancers and precancerous conditions [6]. In addition, the non-cancerous health consequences of chewing BQ are primarily related to the metabolic effects and diabetogenic potential of areca alkaloids. Observational studies have linked BQ use with increased waist circumference [7], obesity [8], hyperglycemia, type 2 diabetes and dyslipidemia [8–10]. The metabolic impairments may propagate intergenerationally through parents who chew BQ habitually [11]. Understanding this intergenerational impact would require an examination of the relationship of BQ chewing during pregnancy with birthweight and postnatal growth as both influence future cardiometabolic risk [12]. While an average reduction in birthweight of 89.5 g (*p* = 0.0028) [13] and a pooled odds ratio of 1.75 for low birthweight (<2500 g) [14] from maternal BQ use have been reported, the effects on growth during early childhood remain unexplored. The biological plausibility of a negative impact of BQ use during pregnancy is supported by mechanistic studies that demonstrated: arecoline crosses placenta [15]; toxicity of arecoline in mice embryos [16]; and growth retardation in arecoline-exposed Zebrafish due to cytotoxicity from depletion of intracellular thiols [17]. Nonetheless, whether the potential growth-retarding effects of using BQ during pregnancy persist into early childhood has not been examined in population-based studies.

The lifetime prevalences of BQ use in south and south-east Asia range from 2.3% to 43.6% [18]. Studies conducted in Bangladesh have documented prevalences of current BQ use of 32.7%–52.8% [19–21]. This high population burden underlines the practice of chewing BQ – unlike smoking or drinking alcohol – being socially acceptable due to long-standing cultural norms [22]. Furthermore, BQ use during pregnancy is considered a means of preventing morning sickness and a relaxant [23]. Unsurprisingly, the prevalence of BQ use among pregnant women can be as high as 61% in rural Bangladesh [24]. In 2021, Bangladesh was the second largest producer of areca nut globally following India, with an annual yield of 345.8 kilotonnes [25]. However, in contrast to maternal dietary behaviors and nutritional deficiencies, potential risks to child growth from maternal BQ chewing received less attention. Stunting and underweight among under-five children in Bangladesh continue to be high (prevalences: 31.1% and 22.5%, respectively) [26], whereas overweight or obesity has started increasing [27]. As these growth abnormalities may influence cardiometabolic risk later in life [28], it is imperative to examine whether maternal BQ use affects child growth in a setting where habitual BQ consumption is common. Hence, we evaluated the associations of BQ use during pregnancy with four indicators of child growth – height-for-age z-score (HAZ), BMI-for-age z-score (BAZ), and fat and fat-free mass – along with potential differences in associations by sex in a rural community of Bangladesh.

## Methods

### Study design, setting and participants

This prospective study drew on the PreSSMat (Preterm and Stillbirth Study, Matlab) birth cohort [29,30] that was set up in the rural area of Matlab, southeast Bangladesh to examine the biological, environmental and socio-demographic predictors of adverse pregnancy outcomes. Matlab is located about 55 km to the southeast of the capital city of Dhaka, and is typical of many low-lying, riverine areas of Bangladesh where agriculture and fishing are the major sources of income [31]. International Centre for Diarrhoeal Disease Research, Bangladesh (icddr,b) has been running a Health and Demographic Surveillance System (HDSS) in Matlab since 1966.

**Figure 1.**
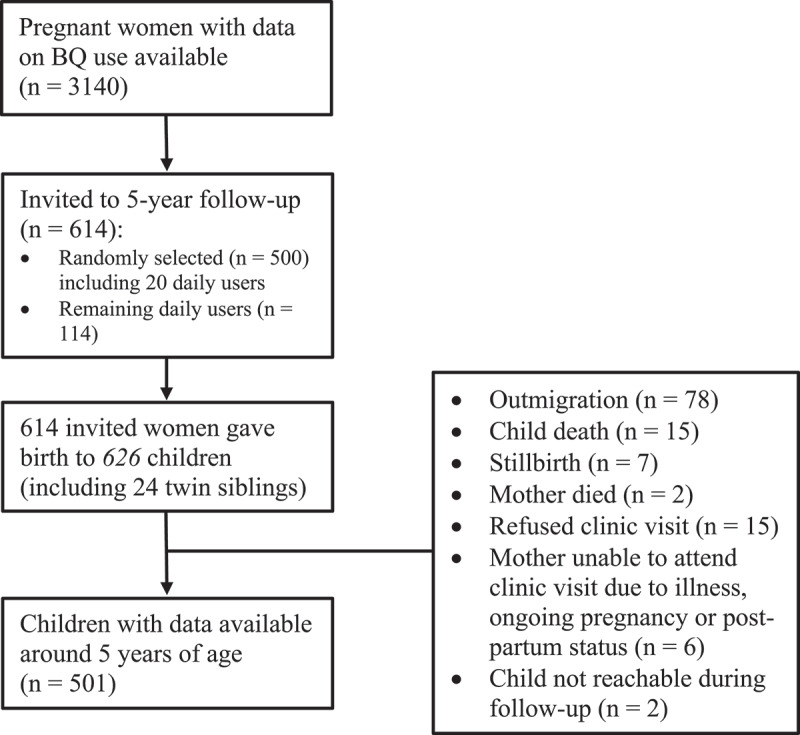
Flow of children into the present study around 5 years of age. BQ, betel quid; GW, gestational week.

PreSSMat utilized the bi-monthly HDSS household visits to identify pregnant women from those with overdue menstrual periods (≥2 weeks) using urine pregnancy tests. The women with a positive test were offered ultrasound to confirm the gestational age and viability of the pregnancy. In total, 3644 women prior to gestational week (GW) 20 were enrolled from May 2015 to June 2017. The enrolled women were followed prospectively till delivery and at 6-weeks post-partum to collect socio-demographic, anthropometric and behavioral data; biological samples; and information on gestational age, pregnancy complications and birth outcomes. Complete data on BQ use was available for 3140 women (median GW at assessment 28, interquartile range: 27–30). Out of these women, children born to a randomly selected subset of 500 women (*that included 20 daily users of BQ*) and to the remaining 114 daily users (*n* = 614) were invited to the growth follow-up between October 2021 and January 2022. Figure 1 at the beginning of the Results section illustrates the flow of participants into the present study.

### Assessment of exposure

A structured questionnaire based on the standard GATS (Global Adult Tobacco Survey) instrument [32] from the World Health Organization (WHO) was employed to assess maternal use of BQ. The enumerators had been trained extensively in the implementation of field survey. According to pre-coded responses, the mothers were grouped as: non-users (did not use BQ in the current pregnancy), previous users (used prior to GW 27–30), current less-than-daily users (used on a less-than-daily basis at GW 27–30), and current daily users (used daily at GW 27–30). For the analysis, the previous and less-than-daily users were categorized together as having ‘intermediate exposure’ and the daily users were labelled as having ‘high exposure’.

### Assessment of outcome

Children’s weight was measured in grams with an electronic scale (Seca 766, 10 gm) and height in centimeters with a portable stadiometer (Seca 214, 0.1 cm). The anthropometric measurements were performed after routine standardization of the technique. The body mass index (BMI) was computed as weight in kilograms divided by height in meters squared (kg/m^2^). Height-for-age (HAZ) and BMI-for-age (BAZ) z-scores were calculated in AnthroPlus [33] using the WHO reference [33]. For descriptive purpose, we categorized the children as thin (BAZ<−2), normal-weight (−2≤ BAZ ≥+1), and overweight or obese (BAZ>+1); and as stunted (HAZ<−2) [34].

We employed leg-to-leg bioelectrical impedance analysis [35] using the tetrapolar Tanita TBF-300A analyzer for assessing body composition. The impedance value from the analyzer reading was extracted and a prediction equation [36] was used to derive fat-free mass (FFM) from the impedance. Khan et al. [36] developed and validated this equation against deuterium oxide dilution (adjusted R^2^ = 0.89; standard error = 0.90; *p* < 0.001) for measuring total body water in a sample of children from Matlab. The values of FFM were subtracted from children’s weight to calculate fat mass.

### Additional variables of interest

Children’s sex (dichotomous: male/female) and age were ascertained during the follow-up. Maternal data extracted from the PreSSMat database included: age, height and weight at enrollment; parity; educational status in terms of completed years of formal education; and household asset score. The asset score was derived by principal component analysis [37] of data on ownership of durable items (e.g. television, almirah, mobile phone, bicycle, etcetera), dwelling characteristics (wall and roof materials), source of drinking water and type of toilet used. A household wealth variable was created by converting the asset scores into tertiles whereby the lowest, intermediate and highest tertiles represented the poorest, middle-status and richest households, respectively. Mothers’ weight prior to GW 20, was measured with a bathroom scale (Seca Uniscale, 100 g) and height with a locally manufactured wooden scale (precision 0.1 cm) [29]. For describing their weight status, mothers were categorized as underweight (BMI <18.5 kg/m^2^), normal-weight (BMI 18.5–24.9 kg/m^2^), and overweight or obese (BMI ≥25 kg/m^2^) [38]. Maternal education was categorized into: primary or below (≤5 years of formal education), secondary (6–10 years), and higher secondary or above (≥11 years). Based on parity, the women were categorized as nulliparous, primiparous, and multiparous (≥2).

### Statistical analysis

Data were analyzed using R statistical software (version 4.3.0) [39]. Visualization of the numerical data involved examination of histograms, boxplots and quantile-quantile plots. Two right-skewed variables were natural log (Ln) transformed: fat and fat-free mass. Children with BAZ>+5 (*n* = 2) were flagged [33] and removed from the respective analyses. At bivariate level, characteristics were compared across exposure categories using Chi-squared test for categorical variables and one-way analysis of variance or Kruskal-Wallis test. We fitted linear regression models – overall and gender-specific – to evaluate the associations of BQ use with the four growth parameters, and reported unstandardized regression coefficients with 95% confidence intervals (CI). We adjusted for children’s sex (when not stratified by it), parity, and maternal height, education and household wealth based on a directed acyclic graph (Supplementary Figure S1). As maternal age and parity had a strong, positive correlation (Spearman’s rho: 0.8, *p* < 0.0001), only parity was included in the model to avoid collinearity. Assumptions of linear regression were checked using quantile-quantile plots of the residuals and residuals versus fitted plots. To examine possible effect modification by child sex, a two-way interaction term (BQ use × child sex) was tested and found non-significant. Statistical significance was set at *p* < 0.05 for all analyses.

### Ethics approval

Research and Ethical Review Committees at https://www.icddrb.org/, Dhaka, Bangladesh approved the study (Protocol#14067; 13 April 2019). Participation was voluntary and participants retained the rights to withdraw throughout. Written informed consent was obtained from the pregnant women before enrollment.

## Results

Figure 1 illustrates the flow of children into the 5-year follow-up. The mothers selected for the follow-up gave birth to 626 children including 12 pairs of twins. Of these 626 children, data from 501 (80%) were available around 5 years of age. The major reasons for loss to follow-up were: outmigration (*n* = 78), parental refusal (*n* = 15) and child death (*n* = 15).

Table 1 presents the basic anthropometric and socio-demographic characteristics of the mothers at enrollment and growth parameters of the children around 5 years of age. Out of the 501 mothers with corresponding data on children’s growth, 217 (43.3%) did not report any BQ use when surveyed at GW 22–24; whereas 177 (35.3%) reported using BQ either in the previous trimester or currently on a less-than-daily basis (‘intermediate exposure’ category) and 107 (21.3%) were using daily (‘high exposure’ category). Median maternal age was higher among daily users than non-users (28.7 versus 25.2 years), and about 47% of the daily users were multi-parous. Nearly 21% of the mothers were overweight/obese at enrollment and the proportion rose to 24.3% among the daily users.

**Table 1.** Maternal and child characteristics.

Characteristics	All (*n* = 501)	Maternal betel quid use		
		Non-user (*n* = 217)	Intermediate^1^ exposure (*n* = 177)	High^2^ exposure (*n* = 107)
**Maternal characteristics at enrollment**				
Age, years	25.1 (21.2–30.4)	25.2 (20.4–28.4)	24.2 (20.4–29.9)	28.7 (23.9–33.6)
Height, cm	151.5 (5.2)	151.7 (4.9)	151.6 (5.1)	151.1 (6.0)
Weight, kg	50.1 (45.1–56.7)	48.9 (44.8–56.3)	50.4 (45.3–56.9)	50.7 (46.0–58.0)
BMI, kg/m^2^	21.7 (19.7–24.3)	21.2 (19.6–24.1)	22.1 (20.1–24.6)	22.3 (20.4–24.8)
**BMI categories**				
Underweight (BMI <18.5)	14.0	14.7	13.0	14.0
Normal (BMI 18.5–24.9)	65.1	65.4	66.7	61.7
Overweight/obese (BMI ≥25)	20.9	19.9	20.3	24.3
**Parity**				
Nulliparous	35.3	40.5	40.1	16.8
Primiparous	34.1	34.6	32.2	36.4
Multiparous (≥2)	30.5	24.9	27.7	46.7
**Maternal education**				
Primary/below (≤5 years)	31.1	24.0	31.6	44.9
Secondary (6–10 years)	57.1	61.7	57.6	46.7
Higher secondary/above (≥11 years)	11.8	14.3	10.7	8.4
**HH wealth**				
Poorest	35.7	38.2	31.6	37.4
Middle-status	34.1	31.8	35.6	36.4
Richest	30.1	30.0	32.8	26.2
**Child characteristics at 5-year follow-up**				
Age, years	4.9 (0.5)	4.9 (0.5)	4.8 (0.5)	4.9 (0.5)
**Sex**				
Female	47.1	43.8	50.8	47.7
Male	52.9	56.2	49.2	52.3
Preterm^**3**^	10.2	10.1	8.5	13.1
Height, cm	103.9 (5.3)	104.2 (5.5)	103.6 (4.6)	103.9 (5.8)
Weight, kg	15.3 (14.0–16.6)	15.4 (14.0–17.3)	15.1 (14.1–16.2)	15.1 (13.8–16.3)
BMI, kg/m^2^	14.3 (13.5–15.1)	14.3 (13.6–15.2)	14.2 (13.5–15.0)	14.2 (13.5–14.9)
HAZ	−1.0 (0.9)	−1.0 (1.0)	−1.0 (0.9)	−1.1 (1.0)
Stunted (HAZ < −2)	14.6	15.2	11.9	17.7
BAZ	−0.7 (1.1)	−0.6 (1.1)	−0.7 (1.1)	−0.7 (1.2)
**BAZ categories**				
Thin (BAZ < −2)	8.6	7.4	9.1	10.4
Normal (−2 ≤ BAZ ≤ +1)	84.2	87.1	83.0	80.2
Overweight/obese (BAZ > +1)	7.2	5.5	7.9	9.4
Fat-free mass, kg	12.6 (11.7–13.7)	12.7 (11.8–13.9)	12.6 (11.7–13.3)	12.6 (11.5–13.8)
Fat mass, kg	2.6 (2.2–3.2)	2.7 (2.2–3.3)	2.6 (2.3–3.0)	2.5 (2.1–3.1)

Abbreviations: BMI, body mass index; HH, household; BAZ, BMI-for-age z-score; HAZ, height-for-age z-score. Values represent percentage for categorical variables; mean with standard deviation for continuous variables (approximately) normally distributed, or median with inter-quartile range for continuous variables that were skewed. ^**1**^Born to mothers who used betel quid prior to, or at gestational weeks 22–24 on a less-than-daily basis; ^**2**^born to mothers who had been daily users of betel quid at gestational weeks 22–24; ^**3**^born before 37 completed weeks of gestation.

Males (52.9%) were over-represented among the children. Approximately 15% of the children were stunted and 7% were overweight/obese. The proportions of both stunting and overweight or obesity were higher among children born to daily users than those born to non-users. There were no differences in children’s fat and fat-free mass levels by BQ exposure categories of the mothers (Table 1).

Table 2 presents the regression coefficients from overall and sex-specific linear regression models analyzing the associations of BQ use with the four outcome variables. None of the regression coefficients were statistically significant. When stratified by sex, female children born to daily users had on average 0.03 standard deviation (SD) lower HAZ, 0.06 SD lower BAZ, 2% lower fat mass and 1% lower fat-free mass than those born to non-users; but the regression coefficients did not attain statistical significance.

**Table 2.** Association of maternal betel nut use during pregnancy with child growth at 5 years.

Parameters	Model	Maternal betel quid use				
		Non-user (*n* = 217)	Intermediate^1^ exposure (*n* = 177)		High^2^ exposure (*n* = 107)	
			β (95% CI)	P	β (95% CI)	P
HAZ	Crude	Ref.	−0.03 (−0.21, 0.16)	0.771	−0.09 (−0.30, 0.12)	0.413
	Adjusted^**3**^	Ref.	−0.02 (−0.19, 0.15)	0.826	0.05 (−0.16, 0.25)	0.641
BAZ	Crude	Ref.	−0.02 (−0.24, 0.20)	0.850	−0.10 (−0.36, 0.15)	0.440
	Adjusted^**3**^	Ref.	−0.02 (−0.23, 0.20)	0.879	0.01 (−0.25, 0.27)	0.949
Ln fat mass	Crude	Ref.	−0.01 (−0.07, 0.05)	0.749	−0.04 (−0.11, 0.03)	0.305
	Adjusted^**3**^	Ref.	−0.02 (−0.08, 0.03)	0.399	−0.01 (−0.08, 0.06)	0.738
Ln fat-free mass	Crude	Ref.	−0.01 (−0.04, 0.01)	0.367	−0.01 (−0.04, 0.02)	0.416
	Adjusted^**3**^	Ref.	−0.01 (−0.03, 0.02)	0.545	0.005 (−0.02, 0.03)	0.756
**Stratified analyses: males**						
HAZ	Crude	Ref.	−0.13 (−0.40, 0.15)	0.375	−0.03 (−0.36, 0.29)	0.831
	Adjusted^**3**^	Ref.	−0.06 (−0.32, 0.19)	0.624	0.18 (−0.13, 0.49)	0.245
BAZ	Crude	Ref.	−0.01 (−0.31, 0.29)	0.948	−0.07 (−0.42, 0.28)	0.692
	Adjusted^**3**^	Ref.	0.01 (−0.29, 0.31)	0.952	0.05 (−0.31, 0.42)	0.772
Ln fat mass	Crude	Ref.	−0.02 (−0.11, 0.07)	0.725	−0.05 (−0.16, 0.05)	0.320
	Adjusted^**3**^	Ref.	−0.005 (−0.09, 0.08)	0.915	−0.002 (−0.11, 0.11)	0.970
Ln fat-free mass	Crude	Ref.	−0.0004 (−0.03, 0.03)	0.983	0.001 (−0.04, 0.04)	0.944
	Adjusted^**3**^	Ref.	0.005 (−0.03, 0.04)	0.759	0.02 (−0.02, 0.06)	0.286
**Stratified analyses: females**						
HAZ	Crude	Ref.	0.05 (−0.20, 0.30)	0.690	−0.10 (−0.40, 0.20)	0.508
	Adjusted^**3**^	Ref.	0.04 (−0.20, 0.29)	0.723	−0.03 (−0.31, 0.26)	0.848
BAZ	Crude	Ref.	−0.002 (−0.32, 0.32)	0.988	−0.11 (−0.49, 0.26)	0.552
	Adjusted^**3**^	Ref.	−0.13 (−0.45, 0.19)	0.420	−0.06 (−0.43, 0.31)	0.739
Ln fat mass	Crude	Ref.	−0.03 (−0.11, 0.05)	0.469	−0.04 (−0.13, 0.05)	0.421
	Adjusted^**3**^	Ref.	−0.06 (−0.13, 0.02)	0.159	−0.02 (−0.11, 0.07)	0.617
Ln fat-free mass	Crude	Ref.	−0.01 (−0.05, 0.02)	0.403	−0.02 (−0.06, 0.02)	0.288
	Adjusted^**3**^	Ref.	−0.03 (−0.06, 0.01)	0.155	−0.01 (−0.06, 0.02)	0.453

Abbreviations: CI, confidence interval; BAZ, body mass index-for-age z-score; HAZ, height-for-age z-score; Ln, natural-log transformed variable where the base of the log was 2.71828. β represents unstandardized regression coefficient. ^**1**^Born to mothers who used betel quid prior to, or at gestational weeks 22–24 on a less-than-daily basis; ^**2**^born to mothers who had been daily users of betel quid at gestational weeks 22–24; ^**3**^Adjusted for children’s sex (when not stratified by it), parity, maternal height and education, and household wealth.

## Discussion

One-fifth of the children were born to mothers who used BQ on a daily basis by the early third trimester, but there was no association between BQ use and child growth around 5 years of age in this cohort study. In addition to the lack of statistical significance, the effect size (based on unstandardized regression coefficients) appeared notably small.

Given that the placenta is not an efficient barrier to arecal alkaloids [15,40] and BQ use during pregnancy may lower birthweight [13], there may have been an attenuation of differences in postnatal growth by maternal BQ exposure. This attenuation could be reflective of a catch-up growth [41] among children born to mothers with higher exposure to BQ. Two aspects related to the daily users of BQ lend support to this proposition. *First*, we observed approximately 108 g (95% CI: 28–189; *p* = 0.008) lower birthweight among the children born to daily users compared to non-users after adjusting for confounders when the full cohort (*n* = 2570) was analyzed (manuscript under preparation). A small proportion of those born to daily users was premature: 14.9% in the full cohort and 13.1% in the present study (Table 1). Longitudinal studies on postnatal growth trajectory associate lower birthweight without prematurity with a catch-up in weight and height starting from 12 months [42,43]. *Second*, nearly a fourth of the daily users in our study were overweight or obese in early pregnancy (Table 1). Maternal overweight or obesity in early pregnancy has been linked to intense catch-up growth and more rapid weight gain among children during the first 3–5 years of life [44,45]. Whereas the catch-up growth may have attenuated the impact of BQ exposure, such a catch-up growth tends to be associated with greater acquisition and more centralized distribution of body fat and insulin resistance later on [41,46–48], leading to a heightening of cardiometabolic risk.

Differences in how exposure was assessed (biomarker assay versus survey data) may affect studies exploring the impact of behavioral exposure – such as use of tobacco, alcohol and caffeine during pregnancy – on postnatal growth. A recent meta-analysis [49] of 21 effect measures from children aged <10 years documented a pooled mean difference of 0.23 kg/m^2^ higher BMI due to maternal smoking during pregnancy. The authors noted that studies ascertaining exposure objectively from serum levels of cotinine – the main metabolite of nicotine – reported higher mean differences in BMI [49]. Conversely, Howe et al. [50] did not find any association between self-reported smoking during pregnancy and children’s BMI change from 2 to 10 years in the ALSPAC cohort from south-east England. Muraro and colleagues [51] followed up a Brazilian birth cohort around 1.5 years (*n* = 2405) and 12 years (*n* = 1716) of age. They did not find any association between smoking during pregnancy (any trimester) and HAZ after controlling for relevant confounders [51]. Among 7597 mother-child dyads of the ALSPAC cohort, no association was observed between self-reported alcohol consumption during pregnancy and children’s height or weight at 2 and 10 years of age despite a negative effect on weight and length at birth [52]. Higher caffeine consumption during pregnancy has been associated with lower HAZ (~0.2 SD between top and bottom quartiles) but not BAZ or fat mass at ages 4–8 years in a cohort study from the US. The investigators ascertained the exposure with blood levels of caffeine and paraxanthine instead of self-reports, yet the magnitude of reduction in HAZ was fairly low [53]. Although influenced by methodological and population-level differences, these findings broadly point toward a recovery in postnatal growth that alleviates the impact of prenatal exposure to these substances to some extent. However, the link between this recovery and future cardiometabolic risk [48] need to be examined for understanding the long-term implications.

The stratified regression analyses did not reveal any notable sex-based differences in the impact of BQ exposure, except that for HAZ as the adjusted regression coefficient was positive for male but negative for female children of daily users (Table 2). Nevertheless, the associations did not attain statistical significance and the interaction terms incorporating children’s sex were not significant as well. The lack of statistical power in stratified analyses should be considered here as there were 51 female and 56 male children born to 107 daily users in the present study. With sample proportions of 43% non-user, 35% intermediate exposure and 21% high exposure, and an alpha of 0.05 (two-tailed); there was 80% power to detect differences of about 0.5 SD across non-user versus high or intermediate exposure categories on sex stratification. Thus, our sample size was unlikely to have captured sex-based differences smaller than that.

Some limitations of the present study are noteworthy. We assessed exposure from responses to survey questions – without measurement of arecoline levels – which may have led to misclassification from recall bias and underestimation of the strength of associations. Residual confounding could not be entirely ruled out in spite of multivariable regression models accounting for confounders identified from a DAG. Attrition from outmigration, refusal to participate and child death contributed to a lowering of statistical power; particularly in stratified analyses. While initially planned, blood samples could not be collected from the children due to general reluctance and refusal by parents to invasive procedures during the early post-COVID period when the follow-up was conducted. Consequently, we were unable to assess any effect of BQ exposure on cardiometabolic markers. The key strengths of the study relate to reliable data from a well-characterized birth cohort in a setting with long-established research infrastructure; rigor in maintaining internal validity; application of a validated equation for bioelectric impedance-based assessment of body composition; and use of simple, cost-effective tools that reduced inter-rater and instrumental biases. The findings are generalizable to under-5 children in Matlab given the area-wide, HDSS-based recruitment of pregnant women in PreSSMat, and also in similar agrarian settings of rural Bangladesh.

## Conclusion

In this prospective analysis, no association was observed between maternal BQ use during pregnancy and child growth in terms of HAZ, BAZ, fat and fat-free mass around five years of age. Catch-up growth among children born to daily users may have played a role in attenuating the impact of BQ use. However, this sort of catch-up growth tends to be associated with future heightening of cardiometabolic risk. Longitudinal studies following up those born to heavy users in adolescence and early adulthood remain key to understanding the implications and cardiometabolic sequelae of any such growth recovery following prenatal exposure to BQ.

## Supplementary Material

Supplementary_material.docx

## Data Availability

Because of the statutory requirements, internal data policies and regulations existing in the collaborating bodies along with the over-arching General Data Protection Regulation (GDPR), the data must be stored in an institutional repository and cannot be made directly accessible without a review of the request for access. Data availability is further limited because the data contain information on gender and health-related and behavioral attributes, and thus, considered to be ‘sensitive personal data’ as per GDPR. Therefore, the data can be accessed only upon formal request that details the purpose of such a request. The request will then be processed by the Data Repository Committee (DRC) at https://www.icddrb.org/. Any such request should be directed to the principal investigators: Professor Eva-Charlotte Ekström(email: lotta.ekstrom@uu.se) and Dr Anisur Rahman (email: arahman@icddrb.org).
