# Supplementary material for: Maternal betel quid use during pregnancy and child growth: a cohort study from rural Bangladesh
Source: Glob Health Action. 2024 Jul 9;17(1):2375829. doi: 10.1080/16549716.2024.2375829 (PMC11234907; doi:10.1080/16549716.2024.2375829)
Supplement: Supplementary_material.docx [file ZGHA_A_2375829_SM1935.docx]

**Supplementary material**

**Supplementary Figure 1.** Directed Acyclic Graph (DAG) showing the pathways (pink color indicates biasing paths requiring confounder controlling) pertinent to the effects of chewing betel nut on postnatal child growth. BN, betel nut; M. edu, Maternal education; HH, household; M., maternal. Prepared using dagitty.net

**Supplementary Table 1.** Unstandardized coefficients from multiple linear regression models analyzing the association of maternal betel nut use during pregnancy with body fat percentage at 5 years

| **Model** | **Maternal betel quid use** | | | | |
| --- | --- | --- | --- | --- | --- |
|  | **Non-user**  **(n = 217)** | **Intermediate^1^ exposure**  **(n = 177)** | | **High^2^ exposure**  **(n = 107)** | |
|  |  | β (95% CI) | P | β (95% CI) | P |
| Crude | Ref. | −0.06 (−0.75, 0.63) | 0.863 | −0.30 (−1.10, 0.49) | 0.454 |
| Adjusted**^3^** | Ref. | −0.33 (−0.90, 0.24) | 0.258 | −0.12 (−0.80, 0.57) | 0.740 |
| **Stratified analyses: males** | | | | | |
| Crude | Ref. | −0.38 (−1.27, 0.50) | 0.396 | −0.57 (−1.59, 0.45) | 0.273 |
| Adjusted**^3^** | Ref. | −0.25 (−1.11, 0.61) | 0.570 | −0.08 (−1.13, 0.97) | 0.879 |
| **Stratified analyses: females** | | | | | |
| Crude | Ref. | −0.20 (−0.97, 0.57) | 0.608 | −0.27 (−1.18, 0.63) | 0.553 |
| Adjusted**^3^** | Ref. | −0.47 (−1.25, 0.30) | 0.230 | −0.14 (−1.03, 0.76) | 0.765 |

CI, confidence interval; β represents unstandardized regression coefficient. **^1^**Born to mothers who used betel quid prior to, or at gestational weeks 22–24 on a less-than-daily basis; **^2^**born to mothers who had been daily users of betel quid at gestational weeks 22–24; **^3^**Adjusted for children’s sex (when not stratified by it), parity, maternal height and education, and household wealth.
